# Time to Cooling Is Associated with Resuscitation Outcomes

**DOI:** 10.1089/ther.2016.0026

**Published:** 2016-12-01

**Authors:** Robert B. Schock, Andreas Janata, W. Frank Peacock, Nathan S. Deal, Sarathi Kalra, Fritz Sterz

**Affiliations:** ^1^ Sid Wolvek Research Center, Life Recovery Systems HD, LLC, Kinnelon, New Jersey.; ^2^ Universitätsklinik für Notfallmedizin, Medizinische Universität Wien, Wien, Austria.; ^3^ Emergency Medicine, Ben Taub General Hospital, Houston, Texas.; ^4^ Baylor College of Medicine, Houston, Texas.

**Keywords:** cardiac arrest, cooling strategies, postresuscitation cooling, human studies, temperature mechanisms

## Abstract

Our purpose was to analyze evidence related to timing of cooling from studies of targeted temperature management (TTM) after return of spontaneous circulation (ROSC) after cardiac arrest and to recommend directions for future therapy optimization. We conducted a preliminary review of studies of both animals and patients treated with post-ROSC TTM and hypothesized that a more rapid cooling strategy in the absence of volume-adding cold infusions would provide improved outcomes in comparison with slower cooling. We defined rapid cooling as the achievement of 34°C within 3.5 hours of ROSC without the use of volume-adding cold infusions, with a ≥3.0°C/hour rate of cooling. Using the PubMed database and a previously published systematic review, we identified clinical studies published from 2002 through 2014 related to TTM. Analysis included studies with time from collapse to ROSC of 20–30 minutes, reporting of time from ROSC to target temperature and rate of patients in ventricular tachycardia or ventricular fibrillation, and hypothermia maintained for 20–24 hours. The use of cardiopulmonary bypass as a cooling method was an exclusion criterion for this analysis. We compared all rapid cooling studies with all slower cooling studies of ≥100 patients. Eleven studies were initially identified for analysis, comprising 4091 patients. Two additional studies totaling 609 patients were added based on availability of unpublished data, bringing the total to 13 studies of 4700 patients. Outcomes for patients, dichotomized into faster and slower cooling approaches, were determined using weighted linear regression using IBM SPSS Statistics software. Rapid cooling without volume-adding cold infusions yielded a higher rate of good neurological recovery than slower cooling methods. Attainment of a temperature below 34°C within 3.5 hours of ROSC and using a cooling rate of more than 3°C/hour appear to be beneficial.

## Introduction

Patient care guidelines from the International Liaison Committee on Resuscitation (ILCOR) and the American Heart Association (AHA) (Donnino *et al.*, 2015) recommend targeted temperature management (TTM) as a treatment for patients who are comatose after return of circulation from cardiac arrest. The newest ILCOR/AHA guidelines recommend cooling to 32–36°C for at least 24 hours to improve the probability of survival with favorable neurological recovery. This represents a change from the prior guidelines (ECC Committee *et al.*, 2010), which recommended a target temperature of 32–34°C for 12–24 hours. The change was largely driven by the “TTM study” (Nielsen *et al.*, 2013), which reported similar outcomes for patients treated with 33°C and 36°C target temperatures. There is some controversy regarding the revised range of target temperatures, and some have suggested that the use of slow, late cooling in the TTM study may have decreased the therapeutic benefit of the 33°C target temperature (Howes *et al.*, 2015; Polderman and Varon, 2015a).

The information that exists regarding optimal timing and depth of postresuscitation TTM is inconsistent. We conducted a meta-analysis of studies with a focus on the impact of early and rapid cooling on neurological outcomes. This analysis was designed after a preliminary review of studies including those discussed hereunder.

## Background

Numerous beneficial effects and their mechanisms of action have been identified for postischemic therapeutic hypothermia, including inhibition of apoptosis and preservation of neuronal integrity (Gonzalez-Ibarra *et al.*, 2011). In one study of pigs subjected to 10 minutes of untreated cardiac arrest followed by 8 minutes of cardiopulmonary resuscitation (CPR), 8/8 (100%) pigs that were rapidly cooled to 32–34°C by surface cooling (∼5°C below the normal temperature of 38°C for this species) within 1 hour of resuscitation and rewarmed 15 hours later fully recovered by 9 days. This compared with 1/8 (12.5%) of animals maintained at normothermia (Janata *et al.*, 2008). Upon necropsy and histological analysis, cooling was associated with a reduction in brain cell damage. Furthermore, blood analysis (White *et al.*, 2006) showed that, although 3 hours after resuscitation arterial neuroprotectin D1 (NPD1) increased in all animals, it was three times higher in hypothermic animals. Unlike NSE and S-100B, which are markers of injury (Calderon *et al.*, 2014), NPD1 is an endogenous lipid mediator that reduces inflammation and apoptosis in neurons undergoing oxidative stress (Eady *et al.*, 2012). These findings suggest that cooling beyond the 3-hour postresuscitation window may miss an important mechanism of neuroprotection.

A similar study found that 7/7 (100%) pigs fully recovered if they were cooled to 32–34°C within 2 hours of return of spontaneous circulation (ROSC) and maintained in that range for 4 hours (Yu *et al.*, 2015). Only 3/7 (43%) control animals that were maintained at normothermia in this study survived 4 days. This study further supported the importance of cooling within the 3-hour post-ROSC window. It also demonstrated that one of the benefits of cooling was an improvement in myocardial function during recovery and that the use of early, rapid cooling may enable the use of a shorter period of therapeutic hypothermia.

Clinical research has provided additional insight into the relationship between earlier cooling and outcomes, although this is complicated by issues related to the methods of cooling. Two randomized studies (Bernard *et al.*, 2010; Kim *et al.*, 2014) investigating prehospital cold intravenous fluid infusions found trends for worsened outcomes, including recurrent cardiac arrest and pulmonary edema, in resuscitated patients (Kim *et al.*, 2014). Other analyses in which a high proportion of patients received cold infusions have likewise failed to demonstrate a benefit of earlier cooling (Nielsen *et al.*, 2009; ICE Study Group, 2012; Debaty *et al.*, 2014), possibly because of complications associated with this approach. The largest of these studies (Nielsen *et al.*, 2009) may have cooled too late to have demonstrated a benefit of earlier cooling (median cooling time from arrest to 34°C was 260 minutes, which exceeds the 3-hour NPD1 window).

Intravenous fluid administration adds venous volume to patients, which can increase preload and decrease coronary perfusion pressure (CPP) (Yannopoulos *et al.*, 2009). Previous work has demonstrated a correlation between CPP and ROSC (Paradis *et al.*, 1990), with decreases associated with failed resuscitation. The observed increased incidence of rearrest associated with cold infusions (Kim *et al.*, 2014) may likewise be related to a reduction in CPP. Therefore, when interpreting studies examining time-dependent outcomes with TTM, the potential for confounding based on the cooling method must be considered.

Comparing trials that achieved their therapeutic hypothermia target of 32–34°C within 2 (Bernard *et al.*, 2002) versus 8 (HACA Study Group, 2002) hours of resuscitation yielded a proportionally higher improvement in outcomes with earlier cooling. One study of 200 postresuscitation patients found that the subset cooled to 34°C within 3 hours had better outcomes than those cooled later (Castrén *et al.*, 2010). Another study of 49 postresuscitation patients found that a 1-hour delay in reaching a target temperature of 33°C was associated with a 31% reduction in favorable neurological recovery (Wolff *et al.*, 2009). Furthermore, a review of 172 resuscitated patients found that every 30-minute delay in reaching 32–34°C was associated with a 17% increase in poor neurological outcomes (Sendelbach *et al.*, 2012), and a related study of 140 patients found that for every 1-hour delay in the initiation of cooling, there was a 20% increase in the risk of death (Mooney *et al.*, 2011). In another study of 171 patients, the best outcomes were achieved when the collapse to 34°C interval was less than 95 minutes (Nagao *et al.*, 2010). A further study of 145 patients (Ubarri *et al.*, 2015) found that patients who reached target temperatures of 32–33°C quickly had a better prognosis than those who were cooled slowly (each 1-minute delay in reaching target temperature reduced the chance of a good outcome by 0.5%).

The advantages of faster achievement of target temperature are most apparent when adjustments are made for comorbidities; patients who have suffered more severe cerebral injury may lose the ability to conserve their own body heat and be more easily cooled (Benz-Woerner *et al.*, 2012; Lin *et al.*, 2014; Perman *et al.*, 2015). A previous study that did not make such adjustments failed to demonstrate a benefit of faster achievement of target temperature (Haugk *et al.*, 2011).

Some trials have suffered flaws that confound interpretation. Two studies (Bernard *et al.*, 2002; HACA Study Group, 2002) allowed controls to have mild fever during recovery (∼37.5°C). This may have worsened results in control patients and overstated the benefits of therapeutic hypothermia (hyperthermia increases ischemia-induced neurological damage) (Hindfelt, 1976).

One study (Lopez-de-Sa *et al.*, 2012) compared 32°C and 34°C target temperatures in 36 postarrest patients. Target temperatures were typically reached 6 hours after resuscitation. The study reported better outcomes in patients who were cooled to 32°C.

The TTM study (Nielsen *et al.*, 2013) compared cooling targets of 33°C with 36°C in 950 patients and reported similar outcomes between cohorts. The results of the TTM study should be viewed with caution (Peacock and Deal, 2014; Howes *et al.*, 2015; Polderman and Varon, 2015a, 2015b). Although both groups were ∼35°C at the time of enrollment (up to 4 hours after resuscitation), the target temperatures were not reached until ∼8 hours later (in the 33°C group, 34°C was reached 2.5 hours postenrollment). Given how late target temperatures were reached, it is possible that the patients did not receive the potential benefits of protective mechanisms that might have been available with earlier cooling to 33°C. If NPD1 release within the first 3 hours after cardiac arrest is necessary, it could be argued that both hypothermia target groups actually had the same level of hypothermia (≈35°C). The animal study by Janata *et al.* (2008) cooled its subjects to ≈5°C below normothermia within the first 3 hours after ROSC, whereas the TTM subjects were only cooled to ≈2°C below normothermia within this window (less than half the “dose” of hypothermia). Earlier cooling might have improved the benefits of the deeper cooling target cohort of the TTM study. This supposition is backed by an analysis (Kaneko *et al.*, 2015) of 467 patients in whom target temperatures were reached within 3 hours of resuscitation; in this study, patients who were rapidly cooled to a temperature range of 32.0–33.5°C (3.5–5°C below normothermia) had significantly better outcomes than those who were rapidly cooled to a range of 34.0–35.0°C (2–3°C below normothermia).

On this basis, we hypothesized that a rapid cooling approach in which patients are cooled to a temperature below 34°C within the first 3.5 hours after resuscitation using a high speed of cooling (to enhance therapeutic effects such as NPD1 release) and avoiding the use of volume-adding cold intravenous infusions (to minimize the harmful effects of increased venous volume) would yield superior outcomes compared with the use of slower cooling approaches with or without cold infusions.

## Methods

### Search strategy

Our meta-analysis examined both randomized and observational studies of the use of therapeutic hypothermia in postresuscitation patients and was conducted following the MOOSE guidelines for meta-analysis of observational studies (Stroup *et al.*, 2000). We performed a systematic review of the literature related to therapeutic hypothermia and resuscitation, as summarized in Appendix 1. This included a search of the PubMed database for studies in English published from January 1, 2012, to January 1, 2015, using the search terms “therapeutic hypothermia” OR “temperature management” AND “cardiac arrest OR resuscitation.” In addition, we examined all of the studies cited in a previous systematic review (Walters *et al.*, 2014). We contacted several authors in an effort to include all available studies.

### Selection of studies for inclusion in meta-analysis

The selection of study inclusion criteria was based on examination of the largest published studies of postresuscitation therapeutic hypothermia. Inclusion criteria included documentation of time delay from collapse to ROSC of 20–30 minutes, reporting of the time delay from ROSC to target temperature, percentage of patients in ventricular tachycardia (VT) or ventricular fibrillation (VF), and therapeutic hypothermia maintained for 20–24 hours. Studies were excluded from numerical analysis if they used cardiopulmonary bypass, did not include time delay from ROSC to target temperature, did not report age, excluded patients >65 years, did not report CPC (Cerebral Performance Categories) scores (Safar, 1981), did not report time interval from collapse to ROSC, did not cool all patients, or excluded patients who died during therapeutic hypothermia.

We sought to analyze results to investigate whether differences in outcomes could be detected between rapid and slower cooling studies. We defined rapid cooling studies as those in which a core temperature of 34°C was reached within 3.5 hours of ROSC without the use of cold volume-adding intravenous infusions, with a ≥3.0°C/hour rate of core body cooling. Because few studies of rapid cooling are available, all are included regardless of size. However, because so many slow cooling studies are available, only slow cooling studies with >100 patients enrolled are included in this analysis.

### Data extraction and analysis

The abstract of each study identified in the search was screened for inclusion/exclusion criteria. If necessary for screening, the full article was reviewed. Study exclusion criteria were identified and tabulated for all citations. Results from included studies were taken directly from each publication and tabulated, including number of patients, time from cardiac arrest to ROSC, patient age, cooling methods, time from ROSC to target temperature, percentage of patients with VT/VF rhythms, and percentage of patients with good outcomes (CPC 1 or 2). Cooling rates in °C/hour were tabulated as reported, or if unreported were calculated from the published study data. Percentages of patients recovering with favorable outcomes were determined for VT/VF and non-VT/VF patients using weighted linear regression analysis (weighted by number of patients in each study) using IBM SPSS Statistics software (Version 22); this analysis was performed for both the rapid and slower cooling study groups. As additional exploratory analyses, we examined speed of cooling and time to target as independent predictors of outcome in post-VT/VF patients.

## Results

**FIG. 1.**
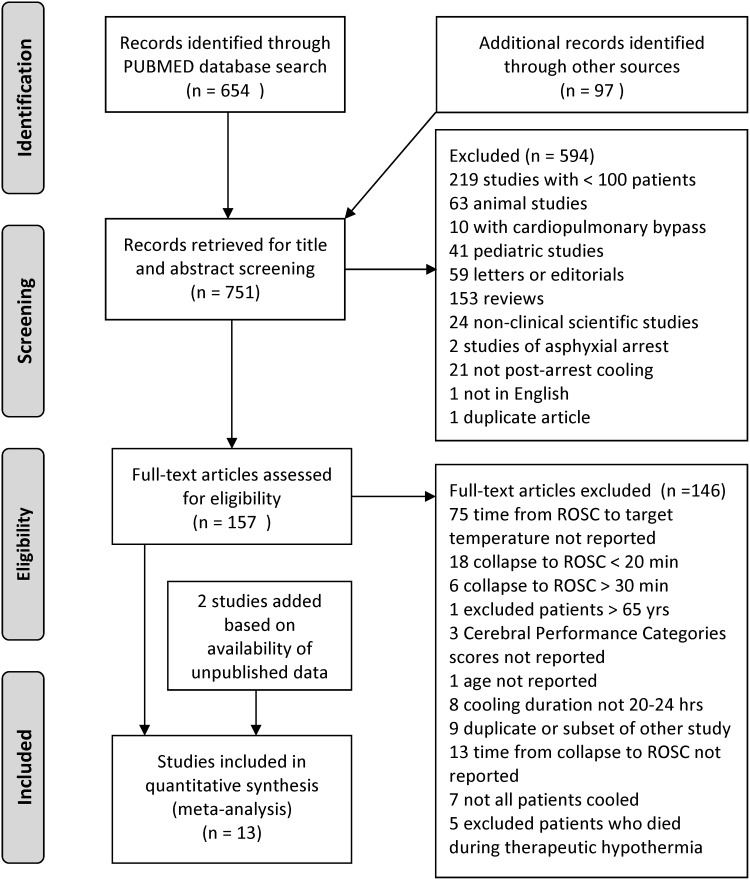
Study selection flow diagram.

Our search strategy, as summarized in Figure 1, identified 751 articles, of which 594 were excluded based on review of abstracts, leaving 157 articles for review. One hundred forty-six of these were excluded based on the criteria shown in Figure 1. Three rapid cooling studies and 8 slower cooling studies comprising 4091 patients were identified by the preliminary search. Two additional studies were added to the analysis after unpublished information was made available by the study authors [these were a slower cooling study (Drennen *et al.*, 2014) and a rapid cooling study (Kudagi *et al.*, 2012)]. This yielded 13 studies comprising a total of 4700 patients. Cooling methods were then dichotomized into relative “speed of cooling cohorts” and consisted of the four clinical trials of rapid cooling with a reported average time from ROSC to target temperature of 2.5 hours and average cooling rate of 4.4°C/hour, and nine slower cooling studies, with a reported average time from ROSC to target temperature of 4.9 hours and average cooling rate of 0.6°C/hour (Table 1). Patient ages in the two cohorts were similar (61.3 and 62.8 years) and the two groups had a similar delay from cardiac arrest to ROSC (23.5 and 22.4 minutes).

**Table 1.** Studies of Rapid Cooling Without Intravenous Cold Saline Infusions (Core Cooling Rate ≥3°C/Hour, 34°C Reached <3.5 Hours Postreturn of Spontaneous Circulation) and Slower Cooling (Core Cooling Rate <3°C/Hour, 34°C Target Reached >3.5 Hours Postreturn of Spontaneous Circulation)

*Author (year)*		*Number of patients*	*Minutes from arrest to ROSC*	*Patient age (years)*	*Cooling methods*	*Time from ROSC to target temperature (hours)*	*Core cooling rate (°C/hour)*	X* = % of population comprising post-VT/VF patients*	Y* = % of patients with good outcomes (CPC 1, 2)*
Howes *et al.* (2010)	VT/VF	15	23	65	Convective immersion surface cooling	3.4	3	100	80
	Non-VT/VF	8						0	38
Kudagi *et al.* (2012)	VT/VF	12	22	59	Convective immersion surface cooling	2.6	3.9	100	83
	Non-VT/VF	21						0	19
Uray *et al.* (2008)		15	26	61	Ice/Graphite pads	1.2	3.3	33	33
Testori *et al.* (2013)		8	26	61	Veno-venous cooling	2.1	12.2	25	38
Overall		79	23.5	61.3	Rapid cooling	2.5	4.4	Results of weighted linear regression: *Y* = 0.582*X* + 0.218; *R*^2^ = 0.936, *p* = *0.002*	
HACA Study Group (2002)		137	21	59	Cold air and ice packs	8.0	0.4	100	55
Arrich *et al.* (2007)		465	23	59	Assorted	4.5	1.1	68	45
Nielsen *et al.* (2009)	VT/VF	686	20	63	Assorted (80% with cold infusions)	4.0	0.7	100	56
	Non-VT/VF	283						0	22
Bernard *et al.* (2010)		234	26	63	Cold infusions and surface cooling	7.5	0.1	100	50
Sendelbach *et al.* (2012)		172	24	64	Ice, gel-faced cooling pads, cooling blankets	4.8	0.9	73	48
Nielsen *et al.* (2013) (33°C cohort)		473	25	64	Assorted	4.5	0.3	79	46
Leary *et al.* (2013)		236	28	58	Assorted	4.0	0.8	32	33
Kim *et al.* (2014)	VT/VF	583	24	62	Assorted, with and without prehospital cold infusions	4.9	0.4	100	60
	Non-VT/VF	776	27	67			0.6	0	14
Drennan *et al.* (2014)	VT/VF	576	22	62	Assorted	6.0	0.5	100	60
Overall		4621	22.4	62.8	Assorted	4.9	0.6	Results of weighted linear regression: *Y* = 0.405*X* + 0.165; *R*^2^ = 0.961, *p* < *0.001*	

The *p* values represent the significance of the ANOVA weighted least squares regression analysis in which Fraction of Good Outcomes vs. Fraction of Shockable Patients was plotted. This number was automatically generated as part of the SPSS program. The very low numbers indicate the high degree of linearity of the data.

CPC, Cerebral Performance Categories; ROSC, return of spontaneous circulation; VF, ventricular fibrillation; VT, ventricular tachycardia.

**FIG. 2.**
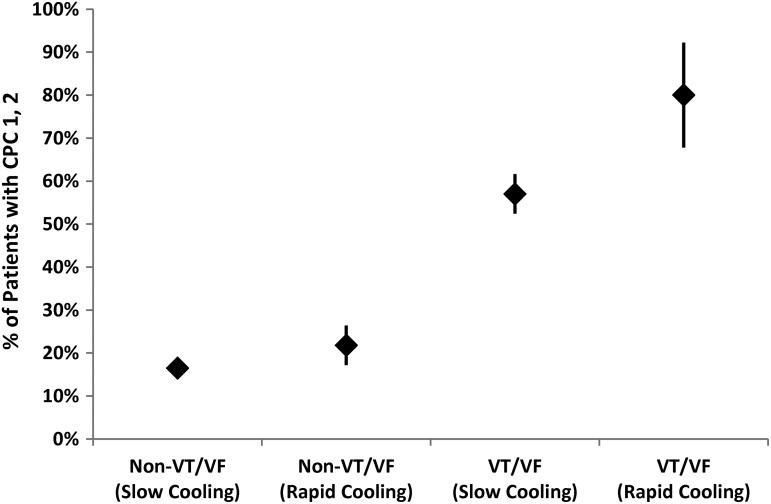
Rates of favorable recovery (CPC 1 or 2) (Safar, 1981) versus Cardiac Arrest Rhythm (Clinical Studies of PostResuscitation Cooling) as predicted by weighted linear regression analysis of clinical studies listed in Table 1 (weighted by number of patients in studies). Standard errors, indicated by the *vertical lines*, do not overlap for VT/VF rhythms, suggesting an advantage of the faster cooling approach for patients resuscitated from shockable rhythms. CPC, Cerebral Performance Categories; VF, ventricular fibrillation; VT, ventricular tachycardia.

**FIG. 3.**
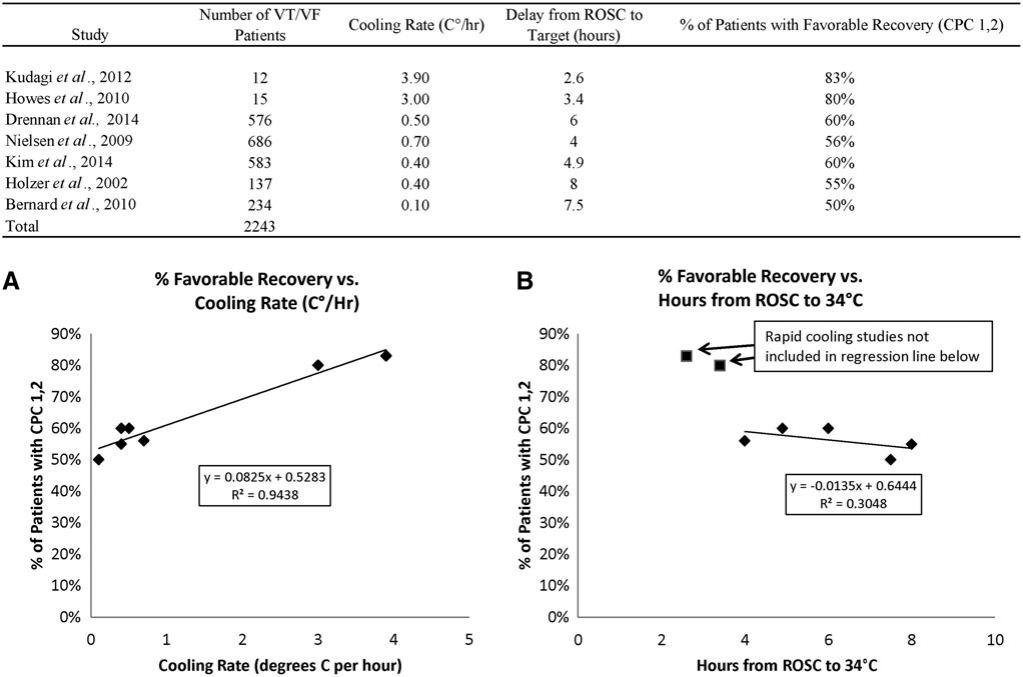
Exploratory linear regression analyses of clinical studies showing chances of good outcomes (CPC 1 or 2) (Safar, 1981) of post-VT/VF patients treated with targeted temperature management versus **(A)** cooling speed (°C/hour) and **(B)** time delay from ROSC to target (hours). *Regression line* in plot **(A)** includes both rapid and slower cooling studies. *Regression line* in plot **(B)** includes only slower cooling studies; faster cooling studies in this plot are shown as *boxes*. ROSC, return of spontaneous circulation.

The outcomes of patients from the rapid cooling studies were superior to those from the slower cooling studies (Fig. 2). The advantage of faster cooling was more pronounced for VT/VF patients than for non-VT/VF patients. Further analysis of the VT/VF patient data suggests that the chances of favorable outcomes for these patients may be linearly related to the rate of cooling (Fig. 3A), with a cooling rate more than 3°C/hour yielding the best results. Time delays from ROSC to 34°C ranging from 4 to 8 hours appear to produce similar outcomes, but a delay of shorter than 3 hours may contribute to improved outcomes (Fig. 3B).

## Discussion

### Summary of evidence

Our meta-analysis examining the time dependency of cooling in 4700 patients suggests that early and rapid cooling without the use of cold infusions is associated with superior outcomes as compared with delayed cooling or cold saline volume resuscitation. Although further prospective studies are needed, our evaluation also suggests that rapid cooling to 32–34°C is associated with superior outcomes as compared with delayed cooling to any temperature. The physiology supporting these findings is consistent with previous basic science laboratory work, early laboratory biomarker investigations, and the confounding associated with cold saline volume resuscitation.

### Risks of bias

A key potential source of bias in the comparison of postresuscitation studies is the influence of cardiac arrest rhythm on outcome. Numerous sources have documented that patients with VT/VF rhythms generally have a better prognosis than those with nonshockable rhythms (Kim *et al.*, 2014; Walters *et al.*, 2014). This makes it challenging to compare studies in which outcomes are not broken down by cardiac rhythms. For this reason, we analyzed outcome data as a function of the percentage of shockable rhythms in each study using a weighted linear regression analysis.

Other potential sources of bias include patient age and delay from collapse to ROSC. Both higher patient age and a longer delay to ROSC are known to worsen outcomes. The studies utilized in our analysis were well matched in these areas, which we believe eliminated the risk of this type of bias.

Our search uncovered only four small studies that met both our definition of rapid cooling and fell within our acceptance criteria, totaling 79 patients. A 200-patient randomized study (Castrén *et al.*, 2010) demonstrated a trend for improved outcomes with earlier cooling (target reached within 2.1 hours of ROSC), but this study employed a cooling rate of only 0.7°C/hour. It was not included in our regression analysis because of its relatively long delay to ROSC (31 minutes). This study reported lower rates of recovery for patients in general (whether treated with rapid or slow cooling) than were seen in comparable studies having shorter delays to ROSC. It has elsewhere been reported that a delay to ROSC of more than 29 minutes reduces the benefits of TTM (Wallmuller *et al.*, 2016).

Two of the rapid cooling studies included in the analysis were conducted using a cooling device marketed by the company that employs one of the authors (R.B.S.). The risk of bias from the use of these studies is judged to be small. One of these studies (Howes *et al.*, 2010) was conducted at three hospitals under Good Clinical Practices guidelines (Food and Drug Administration, 1997), with results audited and verified by an independent clinical research organization. The second of these studies, a single-center study of the same device (Kudagi *et al.*, 2012), was conducted independently by physicians at the Ochsner Research Foundation (New Orleans, LA) without the involvement of the company and produced similar results. The inclusion of this study did not markedly impact the average outcomes for rapid cooling, but it did reduce the standard error.

The disparate sizes of clinical studies could have introduced bias. We sought to minimize this bias by performing an analysis that was weighted by the number of patients in each study. This weighted approach was applied to all key variables, including patient ages, delay to ROSC, and the linear regression analysis of outcomes. We only included studies of 100 or more patients in the slow-cooling group, which may give the appearance of selection bias when compared with the selection of smaller rapid cooling studies. However, exploratory analyses in which we also included smaller studies of slow cooling produced the same line of regression with little change in the standard error. The largest six studies, which totaled 3842 patients, dominated the weighted linear regression results for slow cooling.

### Quality of included studies

The meta-analysis used results from a mixture of medium- to high-quality prospectively randomized studies (HACA Study Group, 2002; Bernard *et al.*, 2010; Nielsen *et al.*, 2013; Kim *et al.*, 2014) and those from nonrandomized studies. Most of the nonrandomized slow-cooling studies corresponded closely to the mean line of regression that was shared with the slow-cooling randomized studies, an observation that supports this approach.

### Impact of cooling rate

A comparison of the effects of cooling rate (Fig. 3A) and time to target (Fig. 3B) suggests that a high cooling rate may be beneficial independently of time from ROSC to target. Rapid cooling induction reduces the length of time during which the patient is exposed to potentially harmful effects of the cooling process, such as metabolic disorders and shivering; shivering is typically reduced at core body temperatures below ∼33.5°C (Polderman and Herold, 2009). A further potential source of cooling-induced physiological stress is nonshivering thermogenesis originating in brown adipose tissue (BAT); this has been reported to cause a 1.8-fold increase in whole-body energy expenditure lasting 3 hours or more in adult humans (Ouellet *et al.*, 2012). Research in anesthetized rats suggests that BAT sympathetic nerve activity and thermogenesis are substantially reduced when core body temperature drops to ∼33°C (Christopher J. Madden, Oregon Health and Science University, Portland, OR, unpublished information). The largest study of prehospital cold infusions (Kim *et al.*, 2014) found that patients receiving prehospital cooling arrived at the hospital at a temperature of 35°C, whereas those not receiving prehospital cooling were nearly 1°C higher. The patients receiving prehospital cooling had statistically lower pH, PaO_2_, SaO_2_, and glucose, suggesting that this treatment created additional, potentially stressful metabolic activity. This may have contributed to the elevated rate of recurrent cardiac arrest in this group. A higher rate of cooling, by dropping temperature more rapidly to a level below 33°C, might reduce the stress of the cooling induction process.

### Impact of time from ROSC to target

Figure 3B shows a nonlinear relationship between outcomes and time to target. The results suggest that outcomes are very similar over the 4–8 hour range of time delay from ROSC to target. A number of studies in which target temperatures were reached beyond the 4 hour window have reported a lack of correlation between outcome and time to target (Nielsen *et al.*, 2009; Kim *et al.*, 2014). This is also consistent with analysis of the TTM study data, which found no signal supporting an advantage of faster cooling (Della Mattia *et al.*, 2015). Our analysis suggests a stepwise improvement in outcomes for times to target below 3 hours, supporting the theory that there is an improved activation of neuroprotective mechanisms if cooling induction is completed within this window. Only a small proportion of patients in the TTM study were cooled to the 33°C level within the 3-hour window (Nielsen *et al.*, 2013).

### Limitations

Our report represents an analysis of multiple studies, and although its size suggests that the identified trends are robust, the presence of significant uncontrolled and unidentified confounders cannot be excluded. Furthermore, the lack of any large rapid cooling investigations may result in an excessive reliance in our analysis on a minority population. Nevertheless, our data suggest that, consistent with many other critical interventions (e.g., glucose for hypoglycemia or oxygen for hypoxia), a time-dependent relationship with outcomes is likely. We suggest that further studies investigating therapeutic hypothermia should report the time interval from the ROSC until target temperature has been reached, as well as the rate of cooling induction.

## Conclusions

The optimal timing and depth of therapeutic hypothermia for postresuscitation patients remain unknown. The value of rapid cooling for victims of cardiac arrest is supported by animal data showing improved outcomes and enhanced release of the neuroprotective lipid mediator NPD1 when rapid cooling is provided within the first 3 hours after resuscitation, and this is further supported by regression analysis of prior clinical studies. Our preliminary analysis suggests that a more rapid rate of cooling may be beneficial in its own right. Analyses of some clinical studies also suggest that deeper cooling may be beneficial, but this is confounded by patient- and treatment-related variables, including the timing of cooling and the use of cold intravenous infusions. We recommend that additional randomized studies be conducted to investigate the impact of rapid cooling treatments on the neurological outcomes of postresuscitation patients. Additional knowledge regarding the value of faster and/or deeper cooling may be gained by measuring the release of NPD1 and other markers of neurological recovery in humans, as well as metabolic indicators related to the physiological stresses of the cooling process.

## Appendix. PUBMED Search Strategy

Search conducted January 21, 2015 on www.ncbi.nlm.nih.gov/pubmed/advanced

All fields: “therapeutic hypothermia” OR “temperature management”

AND All fields: “cardiac arrest” OR “resuscitation”

AND Date of Publication: From January 01, 2012 to January 01, 2015

AND Language: English

Result: 654 citations (included 1 duplicate)

Net: 653 citations available for selection
